# Real‐time FRET assay for monitoring detyrosination by TMCP1 and VASH2

**DOI:** 10.1002/pro.70374

**Published:** 2025-11-12

**Authors:** Matthieu Simon, Julien Espeut, François Juge, Muriel Amblard, Krzysztof Rogowski, Lubomir Vezenkov

**Affiliations:** ^1^ IBMM Université Montpellier, CNRS, ENSCM Montpellier France; ^2^ Tubulin Code Team, Institute of Human Genetics Université Montpellier, CNRS Montpellier France

**Keywords:** Detyrosinase, enzymatic assay, enzyme inhibitor, fluorogenic substrate, FRET, MATCAP, microtubules, peptide, TMCP1, tubulin code, VASH

## Abstract

Tubulin detyrosination is an important α‐tubulin specific posttranslational modification which has been implicated in various disorders including neurodegeneration and cancer. As such, the enzymes involved in the generation of this modification emerged as promising therapeutic targets. Previous studies have identified the members of the vasohibin family, VASH1 and VASH2, as the first class of enzymes involved in the generation of detyrosination. Recently, we have discovered Tubulin MetalloCarboxyPeptidase 1 (TMCP1) as the second class of enzymes catalyzing this modification. Here we describe the development of a highly sensitive FRET‐based enzymatic assay to study and monitor the activity of TMCP1 and VASH2. The originality of this assay lies in the use of 3‐nitrotyrosine as a quencher, which not only restores fluorescence upon cleavage but also closely mimics the natural tyrosine substrate, ensuring optimal enzyme recognition. The selected fluorogenic substrate, named FS2, exhibited strong quenching efficiency and a high signal‐to‐noise ratio, allowing for real‐time kinetic monitoring of TMCP1 and VASH2 activity. Enzyme kinetics, competition assays, and metal ion dependency studies confirmed the assay's specificity, robustness, and physiological relevance. This optimized assay provides a powerful and reliable tool for the future identification and characterization of inhibitors of α‐tubulin detyrosination.

## 1 INTRODUCTION

Microtubules are essential cytoskeletal elements composed of α/β‐tubulin heterodimers that are involved in key cellular processes such as intracellular transport, cell division, and signal transduction. Their dynamic nature and structural versatility not only sustain normal cellular functions but also render them the prime target for therapeutic interventions in various disorders including neurodegeneration (Brunden et al., 2014) and cancer (Čermák et al., 2020). In diseases such as Alzheimer's, microtubule destabilization caused by the detachment of hyperphosphorylated Tau disrupts axonal transport, contributing to neuronal death. Microtubule‐stabilizing agents, such as Epothilone D, help to restore stability and reduce Tau pathology. In cancer, microtubule‐targeting drugs like taxanes stabilize microtubules, while vinca alkaloids prevent their assembly, both treatments resulting in the death of tumor cells. The use of microtubule‐targeting compounds underscores the importance of these cytoskeletal elements as therapeutic targets, although further improvements of these microtubule modulators are needed to enhance specificity, reduce side effects, and overcome resistance in certain cancers.

**FIGURE 1:**
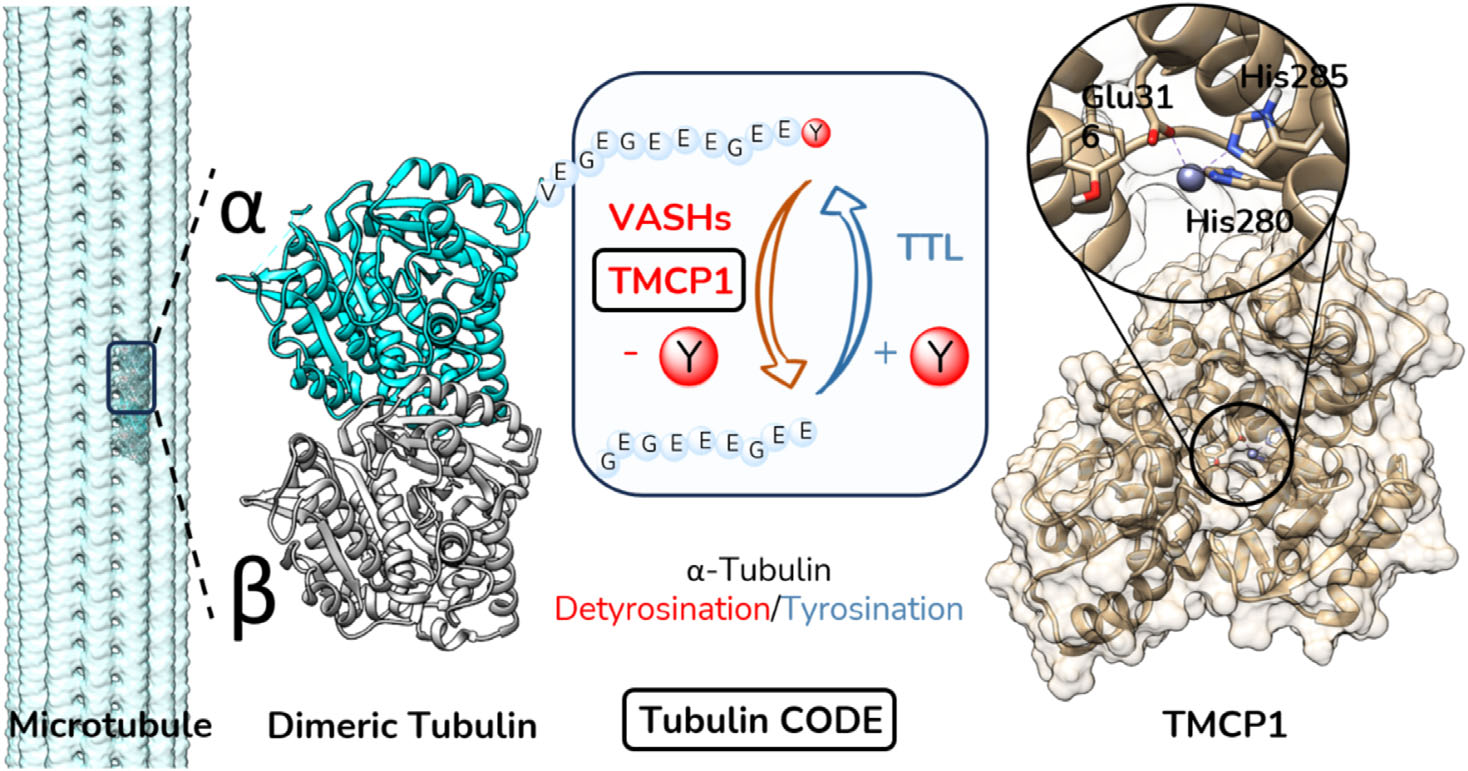
Illustration of the detyrosination/tyrosination cycle of the α‐tubulin C‐terminal tail, along with the enzymes involved, vasohibins (VASHs) and tubulin metallocarboxypeptidase 1 (TMCP1) for detyrosination, and tubulin tyrosine ligase (TTL) for tyrosination Right Panel: The X‐ray crystallographic structure of TMCP1 (PDB: 7Z5H), with a close‐up view of the catalytic site, showing the key amino acids involved in coordinating the metal ion.

Beyond direct microtubule stabilization or destabilization, recent advances have highlighted the importance of tubulin posttranslational modifications (PTMs) such as detyrosination, acetylation, and polyglutamylation in regulating microtubule function (McKenna et al., 2023) (Figure 1). The combination of these modifications together with the expression of various α‐ and β‐tubulin isoforms constitutes a “tubulin code”, which is involved in the functional adaptation of microtubules. As such, the modulation of tubulin PTMs holds the promise of fine‐tuning microtubule behavior in a highly specific manner (Janke & Magiera, 2020). Targeting the enzymes that catalyze these modifications could first help to better understand their role in dynamic biological processes and second pave the way for innovative therapies that precisely regulate microtubule dynamics in both neurodegenerative diseases and cancer, opening the door to a new generation of disease therapies.

The first tubulin modification to be discovered already half a century ago was detyrosination (Hallak et al., 1977). This modification is reversible and consists of the removal of the very C‐terminal tyrosine from α‐tubulin, resulting in the generation of so‐called αΔ1‐tubulin. While the reverse enzyme called Tubulin Tyrosine Ligase (TTL) was identified more than 30 years ago (Ersfeld et al., 1993), the forward enzymes were discovered only recently. First, the two members of the vasohibin family, VASH1 and VASH2, were shown to catalyze tubulin detyrosination (Aillaud et al., 2017; Nieuwenhuis et al., 2017). More recently, we have identified Tubulin MetalloCarboxyPeptidase 1 (TMCP1) (Nicot et al., 2023) also known as MATCAP (Landskron et al., 2022) as the second class of enzymes involved in the generation of this modification.

At the molecular level, the removal of tyrosine alters physicochemical properties of the α‐tubulin C‐terminal tail and as such affects the interactions between microtubules and microtubule‐associated proteins (MAPs) as well as molecular motors (McKenna et al., 2023). Detyrosination was shown to negatively regulate the binding of CAP‐Gly domain‐containing proteins such as Cytoplasmic Linker Protein (CLIP170) and p150^glued^ a member of the dynein/dynactin complex (Peris et al., 2006). It also inhibits the activity of the microtubule depolymerizing motors belonging to the kinesin‐13 family (Corrales et al., 2025; Peris et al., 2009) thus promoting microtubule stability. In contrast, detyrosination also acts as a positive regulator of the binding and activity of various motor proteins including kinesin‐1 (Konishi & Setou, 2009) and kinesin‐7/CENP‐E (Barisic et al., 2015) playing key roles in intracellular transport and chromosome alignment during mitosis. Thus, considering the important role of this modification at the molecular level, it is not surprising that deregulation of detyrosination is associated with various pathologies (Akera et al., 2017; Chang et al., 2002; Chen et al., 2018; Kreitzer et al., 1999; Robison et al., 2016), including neurodegenerative diseases (Barbosa et al., 2017; Aillaud et al., 2017; Iqbal, 2019; McKenney et al., 2016; Nirschl et al., 2016; Pagnamenta et al., 2019; Peris et al., 2022) and cancer (Ferreira et al., 2020; Lavrsen et al., 2023; McKenna et al., 2023; Whipple et al., 2010).

Although VASHs and TMCP1 modify the same substrate, their expression patterns differ significantly across tissues and cell types (VASH1 transcriptomics data: The Human Protein Atlas, n.d.; MATCAP1 transcriptomics data: The Human Protein Atlas, n.d.), suggesting that they play complementary yet distinct biological roles. Thus, to elucidate the function of each class of tubulin detyrosinases, it is crucial to develop selective inhibitors for both types of enzymes.

Considering that we have recently developed highly specific inhibitors for VASHs (Rogowski et al., 2023), the next major challenge is to identify selective TMCP1 inhibitors. A critical step toward this goal is the establishment of an efficient real‐time activity assay, enabling rapid evaluation of potential TMCP1 inhibitors.

Here, we present the development of a highly sensitive Förster resonance energy transfer (FRET)‐based enzymatic assay specifically designed to measure TMCP1 activity. The assay employs a fluorogenic peptide substrate that mimics the C‐terminal sequence of α‐tubulin, TMCP1's natural substrate. This substrate carries a 3‐nitrotyrosine residue (Tyr(3‐NO₂)) in place of the very C‐terminal tyrosine and functions as a quencher of the fluorescent 2‐Aminobenzoic acid (2‐Abz) placed at the N‐terminal side. The cleavage of Tyr(3‐NO₂) by TMCP1 results in the loss of quenching leading to an increase in fluorescence, thus allowing for real‐time kinetic monitoring under various experimental conditions.

As compared to traditionally used immunoblot‐based methods, this assay significantly reduces experimental time and resource consumption, while improving accuracy and reproducibility. Importantly, we further demonstrate that the assay is also compatible with other detyrosinases such as VASH2, thereby broadening its relevance beyond TMCP1. The newly developed assay will greatly facilitate the discovery of TMCP1‐ and VASH‐specific inhibitors. Such probes will be instrumental in elucidating the biological role of tubulin detyrosination. Moreover, the development of these targeted inhibitors could pave the way for novel therapeutic strategies aimed at precise regulation of tubulin posttranslational modifications and consequently microtubule functions.

## 2 MATERIALS AND METHODS

### 2.1 Cloning, production and purification of recombinant protein

#### 2.1.1 TMCP1

Protein production and purification steps were previously described (Nicot et al., 2023). Briefly, TMCP1 open Reading Frame (ORF) was cloned into pET28A+ to produce 6His N‐terminally tagged TMCP1. Transformed Escherichia coli strain BL21 Star (DE3) was grown at 37°C until the optical density (OD600) reached 0.6; then the culture was induced with 0.5 mM isopropyl‐β‐d‐thiogalactoside (IPTG) overnight at 18°C. Cells were pelleted for 15 min at 5000 g and resuspended in a 50 mM Tris–HCl buffer (pH 7.4), 500 mM NaCl, 2 mM TCEP, 0.1% Tween. Cell lysis was performed by sonication followed by centrifugation at 20,000×*g* for 30 min at 4°C. The supernatant was incubated with pre‐washed Ni‐NTA agarose beads (Qiagen) at 4°C for 2 h. Beads were washed extensively in lysis buffer supplemented with 20 mM imidazole and 0.1% Triton. Elutions were performed in a 50 mM Tris–HCl buffer (pH 7.5), 500 mM NaCl, 250 mM imidazole and 1 mM TCEP. The eluted fractions containing proteins were dialyzed overnight at 4°C against 50 mM Tris–HCl buffer (pH 7.5), 300 mM NaCl, 1 mM TCEP using 10 kDa dialysis cassettes. The dialyzed samples were then concentrated and buffer‐exchanged in a 50 mM Tris–HCl buffer (pH 7.5), 200 mM NaCl, 1 mM TCEP using 10 kDa Amicon centrifugal filter units (Amicon Ultra‐4 Merck Millipore). TMCP1 was then snap frozen and stored at −80°C in aliquots.

#### 2.1.2 VASHs

Bicistronic vectors were used to express VASH1 or VASH2 simultaneously with SVBP. The vectors, based on the pET28 backbone, were designed to contain VASH1 or VASH2 tagged at their N‐terminus with His‐ and T7‐tags, upstream from a ribosome‐binding site from T7 phage gene 10, followed by T7‐tagged SVBP. Expression was performed in BL21 Star (DE3) bacteria transformed with the appropriate vector. Protein expression was induced with 0.5 mM isopropyl‐β‐d‐thiogalactoside (IPTG) during 4 h at 37°C. Bacteria were pelleted, washed with PBS, pelleted again and stored at −80° until extraction. The bacterial pellet was resuspended in purification buffer (50 mM Tris pH 8, 300 mM NaCl, 0.05% tween) supplemented with 2 mM TCEP and 10 mM imidazole, and disrupted using a HTU‐DIGI‐F press (Heinemann). His‐tagged proteins were purified using Ni‐NTA agarose beads (Qiagen) in purification buffer supplemented with 20 mM imidazole for washing and with 250 mM imidazole for elution. Fractions containing eluted protein were dialysed in 50 mM Tris, 300 mM NaCl, 10% glycerol, and finally concentrated on Amicon Ultra (Merck). In vitro detyrosination assays were performed with 2 μg Sf9‐derived MTs during 30 min at 37° in 40 μL of buffer (50 mM Tris pH 7.4, 20 μM taxol, 10% glycerol) with the indicated amount of VASH1/SVBP or VASH2/SVBP. The purification of Sf9 tubulin was performed as previously described (PMID: 31851940).

### 2.2 FRET peptide synthesis and purification

All reagents and solvents were obtained from commercial sources and used without further purification. Solid‐phase peptide synthesis was performed on an Iris Biotech® 2‐Chlorotrityl chloride resin loaded at 1.6 mmol/g using Fmoc/t‐Bu chemistry. First, the resin was soaked in Dichloromethane (DCM) for 30 min and filtered. Then, Fmoc‐Tyr(3‐NO_2_)‐OH or Fmoc‐Glu (OtBu)‐OH (0.5 equivalents (eq.)) was solubilized in DCM and a small amount of N,N‐dimethylformamide (DMF) (DCM: DMF, 9:1) and loaded on the resin overnight under agitation with 3 eq. of N,N‐Diisopropylethylamine (DIEA). At the end of this time, the resin was capped with 2 × DCM/Methanol/DIEA (17:2:1) for 10 min, and washed with 3 × DCM; 2 × DMF, 2 × DCM. Then, the deprotection of the Fmoc group at the N‐terminus was performed using a 20% piperidine/DMF solution (2 × 5 min at room temperature). For each coupling reaction, 5 eq. of Fmoc‐Amino Acid, 5 eq. of Hexafluorophosphate Azabenzotriazole Tetramethyl Uronium (HATU) and 10 eq. of DIEA were added to the fritted reaction vessel and stirred in DMF (2 × 5 min at room temperature). After each coupling and deprotection step, the resin was washed three times with DMF. After removal of the Fmoc group of the last amino acid, peptide NS3 was acetylated at the N‐terminus with a DIEA/Ac_2_O/DMF 1/1/8 v/v/v solution (2 × 10 min at room temperature). For fluorescently labeled peptides (FS1, FS2, FS3 and FS2Δ1), 2‐(Boc‐amino) benzoic acid (Boc‐2‐Abz‐OH) was added at the N‐terminus. Peptides were then cleaved from the resin with a TFA/TIS/H_2_O 95/2.5/2.5 v/v/v solution (2 × 90 min at r.t.). The resin was washed (2x DCM) and filtered. The filtrate was evaporated under reduced pressure and then purified by preparative RP‐HPLC on a Waters system controller equipped with a C18 Waters Delta‐Pack column (100 × 40 mm, 100 Å) at a flow rate of 50 mL/min; UV detection at 214 nm was performed using a Waters 486 Tunable Absorbance Detector and a linear gradient of A = H_2_O (0.1% TFA) and B = CH_3_CN (0.1% TFA). Obtained fractions were screened using analytical HPLC analyses on an Agilent Technology 1220 Infinity LC equipped with a Chromolith Speed Rod RP‐C18 185 μm column (50 × 4.6 mm, 5 μm) with a gradient from 100% (H_2_O/TFA 0.1%) to 100% (CH_3_CN/TFA 0.1%) in 5 min; the flow rate was 4 mL/min; detection was at 214 nm. Finally, pure fractions were pooled together and lyophilized (2 times); peptide purity was verified using LC–MS analyses carried out by liquid chromatography (Agilent 1290 Infinity II) coupled to a high‐resolution mass spectrometer (Agilent LC/MSD iQ), equipped with an electrospray ionization source and controlled by OpenLab.

### 2.3 Calibration curve of fluorescent peptides

Calibration curve of the fluorescent peptide substrate was performed by serial dilution (100–0.2 μM) of each peptide FS1, FS2, FS3 and FS2 (Δ1) in a 96‐well plate using a SpectraMax i3x (Molecular Devices) microplate reader. To ensure an accurate estimation of enzymatic activity, the fluorescence of FS2 was subtracted from the measured fluorescence of FS2 (Δ1). Calibration curves were determined for the cleaved peptide FS2 (Δ1) (*Y* = 1,111,206*x*) and the uncleaved peptide FS2 (*Y* = 120,652*x*). The net fluorescence due to the cleaved product was calculated FS2 (Δ1)−FS2 (*Y* = 9,905,554*x*). The quenching efficiency (%) was calculated using the formula: *Q* (%) = 1 − ((FS2)/FS2 (Δ1)) = 89.1%.

### 2.4 Enzyme activity assay

#### 2.4.1 Enzymatic kinetics

For each experiment, a fresh stock solution of the fluorescent peptide substrates (5 mM in DMSO), was prepared and protected from light. TMCP1 was used at a final concentration of 120 nM, obtained by diluting a 60 μM stock solution (stored at −80°C) at a 1:500 ratio. VASH2/SVPB was used at a final concentration of 235 nM, obtained by diluting a 94 μM stock solution (stored at −80°C) at a 1:400 ratio. Enzymatic kinetics experiments were performed in triplicate by measuring the fluorescence of the FRET peptide substrates cleaved by the two enzymes. The cleavage releases a nitro‐tyrosine residue, which acts as a quencher. Its removal leads to an increase of fluorescence, which was quantified fluorometrically (Ex/Em = 320/420 nm) using a SpectraMax i3x (Molecular Devices) microplate reader. After corresponding blank subtraction, enzyme activity in different conditions was calculated using this equation:

$$\text{Enzyme activity}\;\left(\%\right)=\begin{array}{l} 100-(\left(\text{SlopeEC}–\text{SlopeSC}\right)/\text{SlopeEC}\;\times \;100) \end{array}$$

Where Slope SC is the slope (RFU/min) of the sample under altered conditions (pH, EDTA, NS3, Metal ions, NaCl, inhibitors and different buffer) and Slope EC is the slope of the enzyme control, corresponding in this study to 100% enzyme activity (100 μM FS2, with 120 nM of TMCP1 or 235 nM of VASH2/SVPB in a 50 mM Tris–HCl buffer (pH 7.4) supplemented by 10% Glycerol at 37°C).

Data were analyzed using GraphPad Prism, with a dedicated curve fitting applied. A Gaussian bell‐shaped curve was used for the optimal pH study, and dose–response inhibition curves were generated by plotting the concentration of NaCl, EDTA, NS3, etc., against the normalized initial velocity (Vi) corresponding to enzyme activity. Data were normalized so that the curve ranged from 100 to 0% activity.

Optimal pH was established using a 50 mM Tris–HCl buffer, with the pH adjusted to the desired value (ranging 4–11) using 0.1 M NaOH or HCl solutions. For the ionic strength experiments, a 500 mM NaCl stock solution in a 50 mM Tris–HCl buffer was used as a starting point for serial dilution (500–0 mM). For EDTA analysis, a 10 mM stock solution was prepared and fully solubilized by vigorous stirring at 80°C. EDTA (0.004 to 400 μM) was then incubated with TMCP1, for 30 min at 37°C before adding the fluorescent substrate and starting the fluorescence measurement. Subsequently, an identical experiment was conducted using 2‐fold serial dilutions to refine the curve around the previously determined IC_50_.

For VASH2–SVBP, a similar dose–response protocol was applied for the covalent inhibitors LV80 and EpoY, starting from 10 mM DMSO stock solutions diluted in Tris–HCl buffer. Serial dilutions were prepared prior to enzyme addition (235 nM final concentration) to minimize time‐dependent inhibition effects and to avoid premature covalent modification of the enzyme during the dilution steps. All other conditions, including buffer composition, incubation time, and fluorescent substrate concentration, were identical to those described above.

For metal ion dependency assays, the enzyme was first incubated with 50 μM of EDTA for 10 min at room temperature. Then, serial dilutions were performed from 100 mM stock solutions of each metal ion (ZnCl₂, CoCl₂, and MnCl₂.). After a 30‐min incubation at 37°C in the microplate reader, FS2 was added to initiate the reaction.

Finally, the competition assay was performed similarly, except that the 96‐well plate was kept on ice during the serial dilution of the NS3 substrate and its addition into the wells. The incubation step was omitted, and FS2 was added directly afterward to start the reaction.

#### 2.4.2 *Enzyme characterization (*V*_max_,* K_m_ *,* k*_cat_ and* k*_cap_)*

*V*_max_ and *K*_*m*_ were determined by calculating the initial velocity at different FRET peptide concentrations (1 μM–200 μM). Then, using GraphPad Prism and the equation model of Michaelis–Menten (*Y* = *V*_max_*X/(*K*_*m*_ + *X*)), *K*_*m*_ and *V*_max_ were obtained. *V*_max_ was then converted to μM.sec^−1^ using the calibration curve obtained previously (FS2 (Δ1)‐FS2). Then the turnover number (*k*_cat_) was calculated using the following equation: *K*_cat_ = *V*_max_/[*E*]*t* where [*E*] is the total enzyme concentration. Finally, the catalytic efficiency (*k*_cap_) of the enzyme was obtained by dividing the *k*_cat_ by the *K*_*m*_.

## 3 RESULTS AND DISCUSSION

Having confirmed previous results showing that TMCP1 has the ability to cleave peptides corresponding to the last 12 amino acids of α‐tubulin's C‐terminus (Landskron et al., 2022), we aimed at identifying a fluorogenic peptide substrate for monitoring the activity of this enzyme. If successful, it would allow for the development of an easy‐to‐handle enzymatic assay to screen for TMCP1 inhibitors. The use of fluorogenic substrates for carboxypeptidases (CPs) remains limited as compared to endopeptidases (Kuriki et al., 2018; Liu et al., 2017; Yoo & Han, 2021). In the case of endopeptidases, substrate recognition typically occurs upstream of the cleavage site, enabling straightforward fluorogenic substrate design. In contrast, CPs recognize the C‐terminal residue, with cleavage occurring just before the recognition site. For example, a common strategy for endopeptidases consists of attaching fluorogenic moieties, such as 7‐amino‐4‐methylcoumarin (AMC), to the C‐terminus (Breidenbach et al., 2020). Such a strategy, however, could be difficult to apply for CPs because they are highly specific for the C‐terminal residue and the presence of a bulky, non‐natural group like AMC at this position could interfere with substrate recognition and enzymatic activity. Different approaches can be envisioned to overcome this problem, including the use of modified fluorophores with cleavable linkers (Vezenkov et al., 2015), the selection of a small fluorophore to minimize steric hindrance and ensure proper access to the enzyme's active site (Chyan et al., 2017), or the design of a FRET system in which fluorescence is restored upon enzymatic cleavage (Rodriguez‐Rios et al., 2022). However, developing a FRET substrate for a carboxypeptidase such as TMCP1 is challenging, as the donor or acceptor molecule must replace the very C‐terminal residue recognized by this type of enzyme. In some FRET peptides, an acceptor that acts as a quencher, such as 2,4‐dinitrophenyl or more notably 3‐nitrotyrosine, can fulfill this function. These compounds closely mimic tyrosine, the natural amino acid recognized by TMCP1, making them well‐suited for potential recognition and cleavage. Moreover, 3‐nitrotyrosine is commercially available as a Fmoc protected amino acid, enabling its incorporation into peptides via standard Fmoc/tBu solid‐phase peptide synthesis protocols.

**FIGURE 2:**
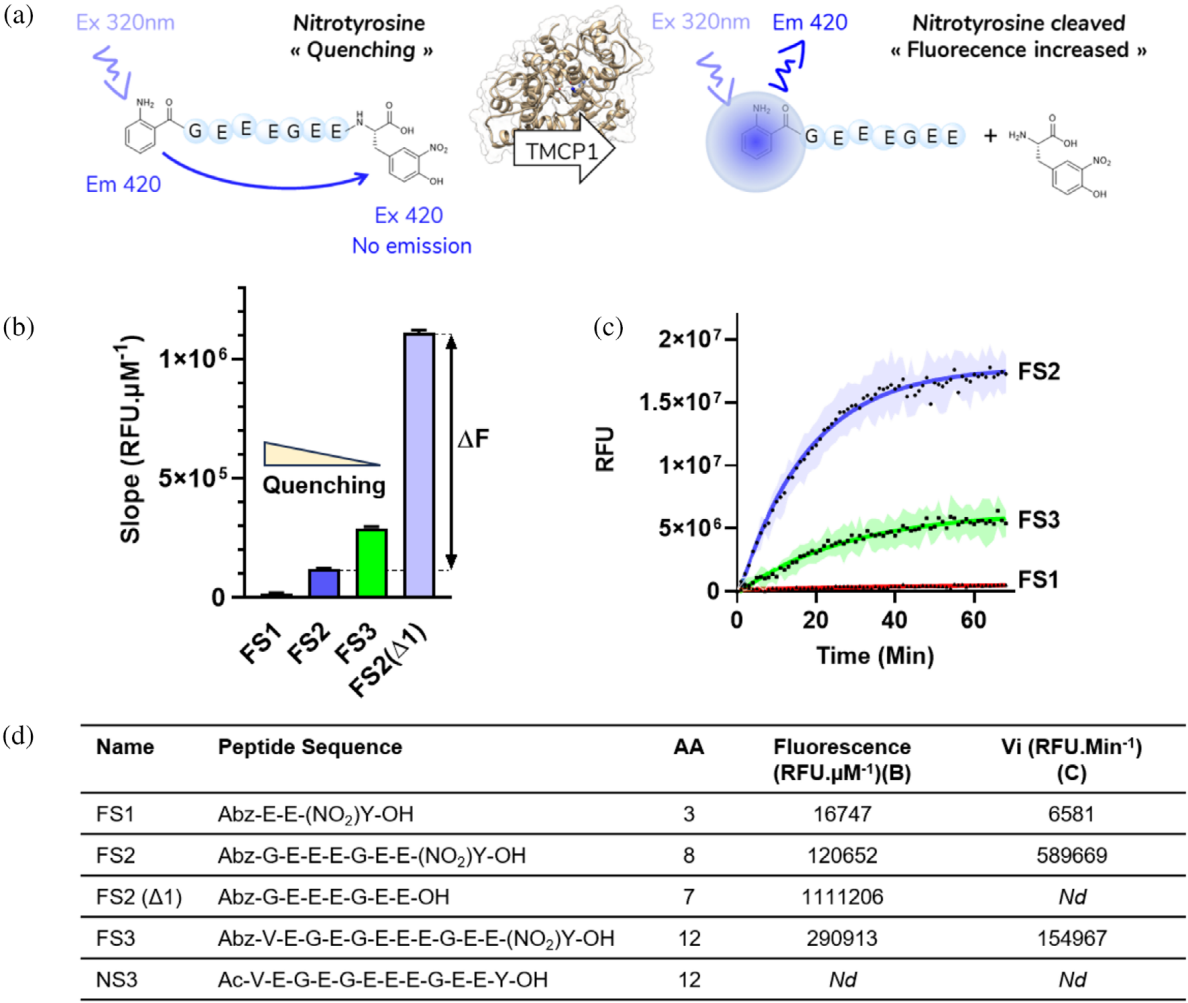
(a) Schematic representation of the enzymatic assay: Upon cleavage by TMCP1 (of the target sequence FS2, as an example), the quencher is released, resulting in an increase in fluorescence. (b) Slope of the peptide's fluorescence calibration curve (mean ± SEM, *n* = 3). ΔF corresponds to the difference in fluorescence between the cleaved and non‐cleaved FS2 peptide. (c) Kinetic experiment using 125 μM of each fluorescent peptide (FS1–3) with 120 nM of TMCP1 (mean ± SEM, *n* = 3). Corresponding values of the peptide fluorescence calibration slopes and Initial velocity (Vi) are reported in table D. (d) Summary table of synthesized peptides: FS (Fluorogenic Substrate), NS (Natural Substrate), AA corresponds to the number of amino acid residues.

Based on these considerations, we selected the 2‐Aminobenzoyl (Abz) as a donor positioned at the N‐terminal part of the potential FRET substrates and 3‐nitrotyrosine as a quencher positioned at the C‐terminal part (Figure 2a). This combination of donor/quencher pair has been previously used to monitor endopeptidase activity (Breddam & Meldal, 1992). The length of the peptide mimicking the natural substrate of TMCP1 (Figure 1) is a critical factor that should ensure both effective quenching and successful recognition by TMCP1 (Landskron et al., 2022). To explore these parameters, three peptides of different lengths were synthesized: a 12‐amino‐acid sequence (FS3), and two shorter variants comprising 8 (FS2) and 3 amino acids (FS1), respectively (Figure 2d).

First, the fluorescence of the synthetized peptides was assessed by performing calibration curve at different concentrations of the peptides (Figure 2b). The obtained results indicate that the short peptide (FS1, 16,747 RFU μM^−1^) exhibited the strongest quenching, as its residual fluorescence was the lowest, suggesting an optimal FRET efficiency resulting from close proximity between Abz and Nitro‐tyrosine. In contrast, the longest peptide (FS3, 290,913 RFU μM^−1^) showed significantly higher fluorescence, suggesting a moderate quenching, likely due to an increase in the distance between the donor and acceptor. The medium‐length peptide (FS2, 120,652 RFU μM^−1^) displayed an intermediate fluorescence value, indicating a moderate but effective quenching efficiency.

A preliminary kinetic experiment was conducted using fixed concentrations of fluorescent substrates (125 μM) and TMCP1 enzyme (120 nM), monitoring enzymatic activity through fluorescence increase upon quencher release (Figure 2c). FS1 was not processed by the enzyme, most likely due to its length, being too short for efficient recognition as a substrate. In contrast, FS2 and FS3 were cleaved by the enzyme over time, reaching a plateau after 30 min. Notably, the fluorescence increase resulting from the cleavage of the nitro tyrosine residue was over three times higher for FS2 as compared to FS3. The cleavage of FS2 into FS2Δ1 and free 3‐nitrotyrosine by TMCP1 was further confirmed by LC–MS, providing a non‐fluorescent validation of the enzymatic activity (Figure S1). To generate an effective read‐out for the enzymatic activity, FRET peptide‐based substrates must be efficiently processed and produce the highest possible fluorescence change (Δ*F*). Based on these requirements, FS2 was selected over FS3 for the follow‐up experiments in this study. Moreover, the synthesis of this peptide was easier and more cost‐effective. To accurately quantify the amount of cleaved peptide during the enzymatic assay, detyrosinated FS2 peptide was synthesized and used to generate a calibration curve (Figure 2b). The difference in fluorescence between the cleaved or non‐cleaved peptide slopes (Δ*F*) was used as a conversion factor, enabling fluorescence changes to be translated into reaction rates expressed in micromoles per second (μM s^−1^). Such representation ensured that the kinetic parameters are expressed in absolute concentration units, providing a more precise quantification of the enzymatic activity. These calibration curves also allowed determination of the quenching efficiency, which reached 89.1% for the FS2 peptide.

**FIGURE 3:**
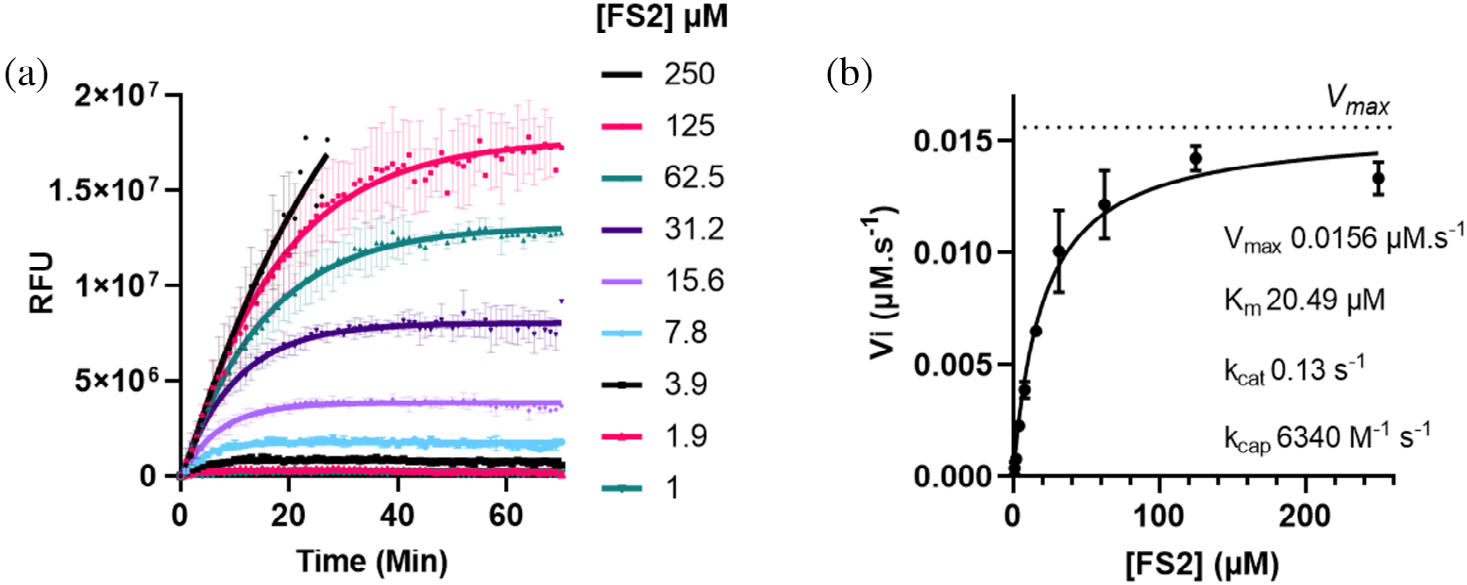
Enzyme kinetics of TMCP1 using FS2 as a fluorescent substrate. (a) Time‐course fluorescence measurements at decreasing substrate concentrations (250–1 μM), showing the enzymatic reaction progression (mean ± SEM, *n* = 3). (b) Initial reaction velocities (Vi) plotted against substrate concentration, fitted to the Michaelis–Menten equation using GraphPad Prism to determine kinetic parameters.

To determine the enzymatic kinetic parameters, a fluorescence‐based assay was performed using FS2 as a FRET peptide substrate. The reaction was monitored in real‐time by measuring the increase in fluorescence upon substrate cleavage. A range of substrate concentrations (from 1 to 250 μM) was incubated with 120 nM of TMCP1 at 37°C in a 50 mM Tris HCl buffer (pH 7.4) supplemented with 10% glycerol (Figure 3a). Fluorescence signals were recorded at regular intervals to follow the reaction's kinetics progression. Initial velocities (Vi) were determined from the linear phase of the reaction, and a Michaelis–Menten curve was generated by plotting Vi as a function of substrate concentration (Figure 3b).

Such analysis allowed us to determine the key kinetic parameters, including the Michaelis constant (*K*_*m*_ = 20.49 μM) and the maximum reaction velocity (*V*_max_ = 0.0156 μM s^−1^). Based on the *K*_*m*_ value, the substrate concentration for subsequent experiments was set at 100 μM, corresponding to approximately five times the *K*_*m*_. This ensures that the enzyme operates under saturating conditions (at or near its *V*_max_). As such, it minimizes the influence of limited substrate availability on the reaction rate, allowing for a more accurate measurement of the enzyme's maximum catalytic activity. All parameters are compiled in Figure 3b, including the catalytic constant (*k*_cat_ = 0.13 s^−1^), which represents the number of substrate molecules converted per second under saturating substrate conditions, providing a measure of the enzyme's efficiency. Additionally, the catalytic efficiency was calculated (*k*_cat_/*K*_*m*_ = *k*_cap_ = 6340 M^−1^ s^−1^) to assess the enzyme's catalytic efficiency, reflecting both substrate binding and conversion.

Although the experimental setup described above is suitable for conducting the enzymatic assay, exploring both optimal and limiting conditions can yield further valuable insights. Systematic screening of factors such as ionic strength, buffer composition, and pH can help to identify conditions that enhance or inhibit the enzymatic activity of TMCP1. Such optimization not only strengthens the robustness, reproducibility, and compatibility of the assay across different experiments but also provides precise analysis of the biochemical properties of the enzyme (Bisswanger, 2014). Given that TMCP1 was only recently discovered, further characterization of its activity under different conditions will contribute to a better understanding of its catalytic mechanisms, stability, and potential applications.

**FIGURE 4:**
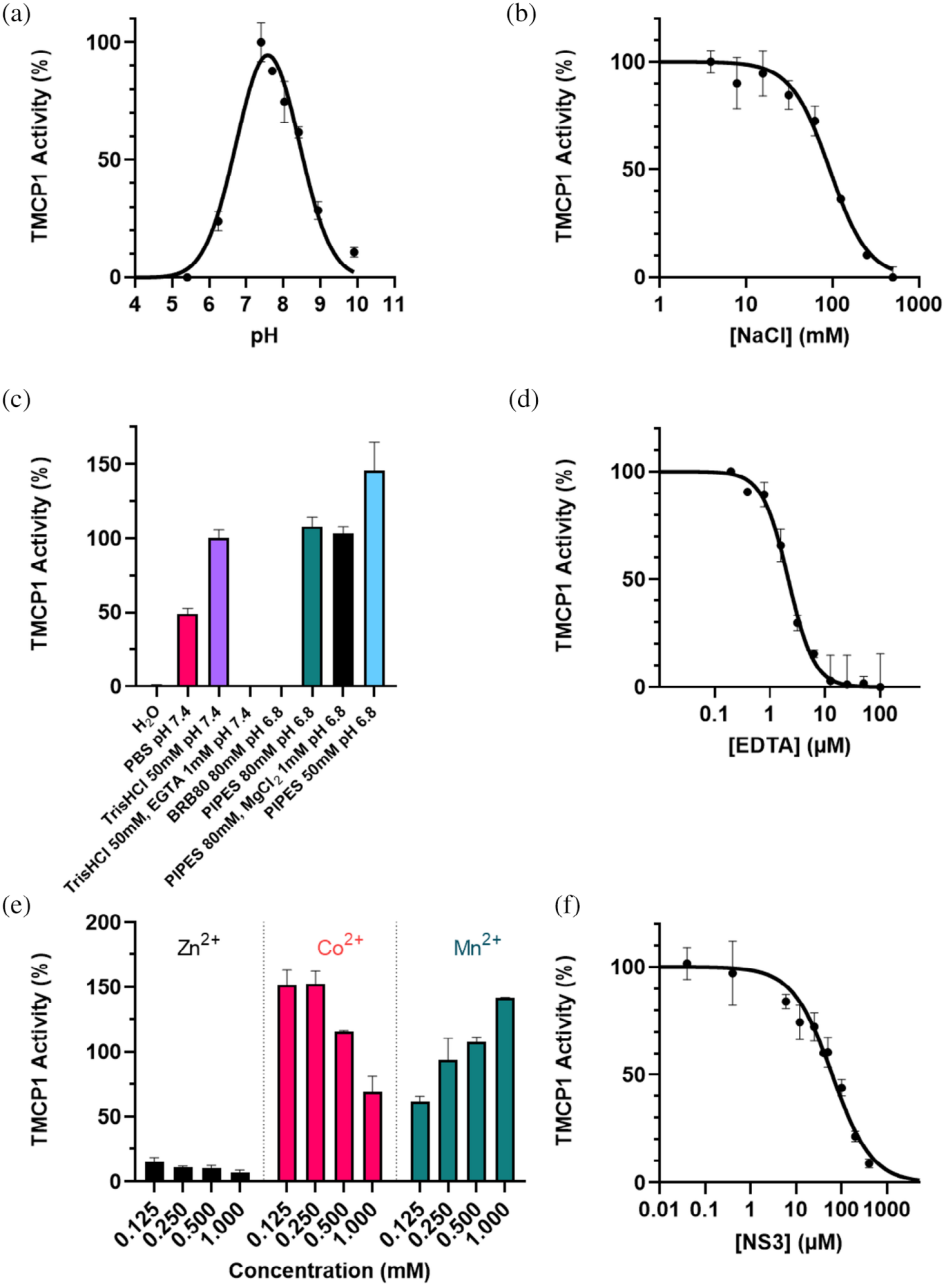
Characterization of TMCP1 enzymatic activity under various conditions (Data are presented as mean ± SEM, *n* = 3 for all experiments). (a) pH dependence of TMCP1 activity, with an optimal activity around pH 7.5. (b) Effect of NaCl concentration on TMCP1 activity, showing almost complete inhibition at NaCl concentrations above 200 mM. (c) Influence of different buffer systems on TMCP1 activity, highlighting variations depending on buffer composition and pH. Composition of PBS is: 137 mM NaCl, 2.7 mM KCl, 10 mM Na₂HPO₄, and 1.8 mM KH₂PO₄. and composition of BRB80 is: 80 mM PIPES (pH 6.8), 1 mM MgCl₂, and 1 mM EGTA. (d) Inhibition of TMCP1 by EDTA, indicating its dependence on divalent metal ions (IC_50_: 2.15 μM). (e) Effect of divalent metal ions on TMCP1 activity. Please note that Zn^2+^ inhibits the enzyme, while Co^2+^ and Mn^2+^ enhance its activity in a concentration‐dependent manner. (f) Dose–response inhibition curve of TMCP1 by NS3.

First, the evaluation of the optimal pH was performed (Figure 4a), demonstrating that TMCP1 shows maximal activity close to a physiological pH (around 7.5). These results are consistent with the enzyme being active primarily in the cytosol.

The impact of increased ionic strength was also evaluated (Figure 4b). TMCP1 exhibited a 50% reduction in activity when the NaCl concentration reached around 100 mM. The affinity of the FRET peptide for TMCP1 is mainly driven by electrostatic interaction between the acidic glutamate residues of the peptide and the cationic arginine and lysine residues of TMCP1 (Landskron et al., 2022). Thus, a significant increase in ionic strength is expected to interfere with enzyme–substrate interactions, leading to reduced catalytic efficiency. This finding is consistent with buffer screening results, where a similar 50% reduction in TMCP1 activity was observed in a high ionic strength buffer such as PBS (137 mM NaCl) (Figure 4c). For enzymes that are sensitive to ionic strength like TMCP1, using buffers with lower or more controlled salt concentrations, like 50 mM Tris–HCl or 50 mM PIPES leads to an improvement in the activity. However, complete absence of salt, like in pure deionized water, results in complete loss of TMCP1 activity. These results highlight the necessity of a buffer to stabilize the pH and maintain ionic strength, both of which are essential for metal ion binding and enzyme function. Finally, no activity was detected in BRB80 buffer, which is considered as the golden standard in the microtubule field. To determine which component of BRB80 was responsible for this lack of activity, additional experiments were performed in 80 mM PIPES, with or without 1 mM MgCl₂, yielding enzyme activity comparable to those obtained in 50 mM Tris–HCl. A complementary experiment in which Tris–HCl buffer was supplemented with 1 mM EGTA resulted in a complete loss of enzymatic activity. This clearly indicates that the lack of activity of TMCP1 in BRB80 buffer can be attributed to the presence of 1 mM EGTA, which, similarly to EDTA, is a chelating agent that binds divalent metal ions present in the active site of TMCP1.

Next, EDTA was used as a control inhibitor to verify the reliability of the assay for drug screening development (Figure 4d). As previously mentioned, EDTA inhibits metalloproteases by chelating their essential metal cofactors, thereby rendering the enzyme catalytically inactive. Although EDTA is highly effective, its inhibition lacks specificity, as it can also affect other metalloproteins. In our assay, an IC_50_ value of 2.15 μM was determined for EDTA. This result is in agreement with a previous study, which reported an IC_50_ between 5 and 10 μM by immunoblotting using GST‐α‐tubulin as a substrate (Nicot et al., 2023).

In addition, considering that TMCP1 is a metallocarboxypeptidase, its metal ion dependency was also assessed (Figure 4e). First, the metal ions present in the active site of TMCP1 were chelated with 50 μM EDTA. Subsequently, ZnCl₂, MnCl₂, or CoCl₂ were added at varying concentrations (>50 μM) to determine their impact on the restoration of enzymatic activity. In agreement with previous findings (Nicot et al., 2023), the addition of ZnCl₂, did not restore the enzymatic activity following the treatment with 50 μM EDTA. In contrast, CoCl₂ was highly effective not only at restoring but even increasing TMCP1 activity, although its effectiveness was reduced at higher concentrations. Finally, MnCl₂ also restored the activity but unlike CoCl₂, it did so in a concentration‐dependent manner. These results are consistent with previous immunoblot‐based assays using tubulin as a substrate, in which TMCP1 was strongly activated by Co^2+^ and Mn^2+^, but inhibited by Zn^2+^ (Nicot et al., 2023). Furthermore, these data suggest that despite the presence of Zn^2+^ in the active site of TMCP1 in the high‐resolution X‐ray structure of this enzyme (Landskron et al., 2022), it is unlikely to represent the actual metal ion bound by the enzyme under physiological conditions (Nicot et al., 2023). One possible explanation is that Zn^2+^ ions were introduced into the active site by the buffer conditions used for the crystallization experiments, which contained a high concentration of this metal (Landskron et al., 2022).

Subsequently, fluorescent substrate cleavage was assessed in the presence of a competing, non‐fluorescent peptide mimicking the last 12 amino acids of the C‐terminal tail of the α‐tubulin (NS3) (Figure 4f). In this assay, the natural substrate exhibited an IC₅₀ of approximately 62 μM, while the fluorescent peptide was used at 100 μM. These results clearly indicate that both peptides compete for the same active site, with a slightly higher affinity for the natural substrate. Overall, these findings validate the specificity of the assay and support its use for further kinetic analyses and competitive inhibitor screening studies.

**FIGURE 5:**
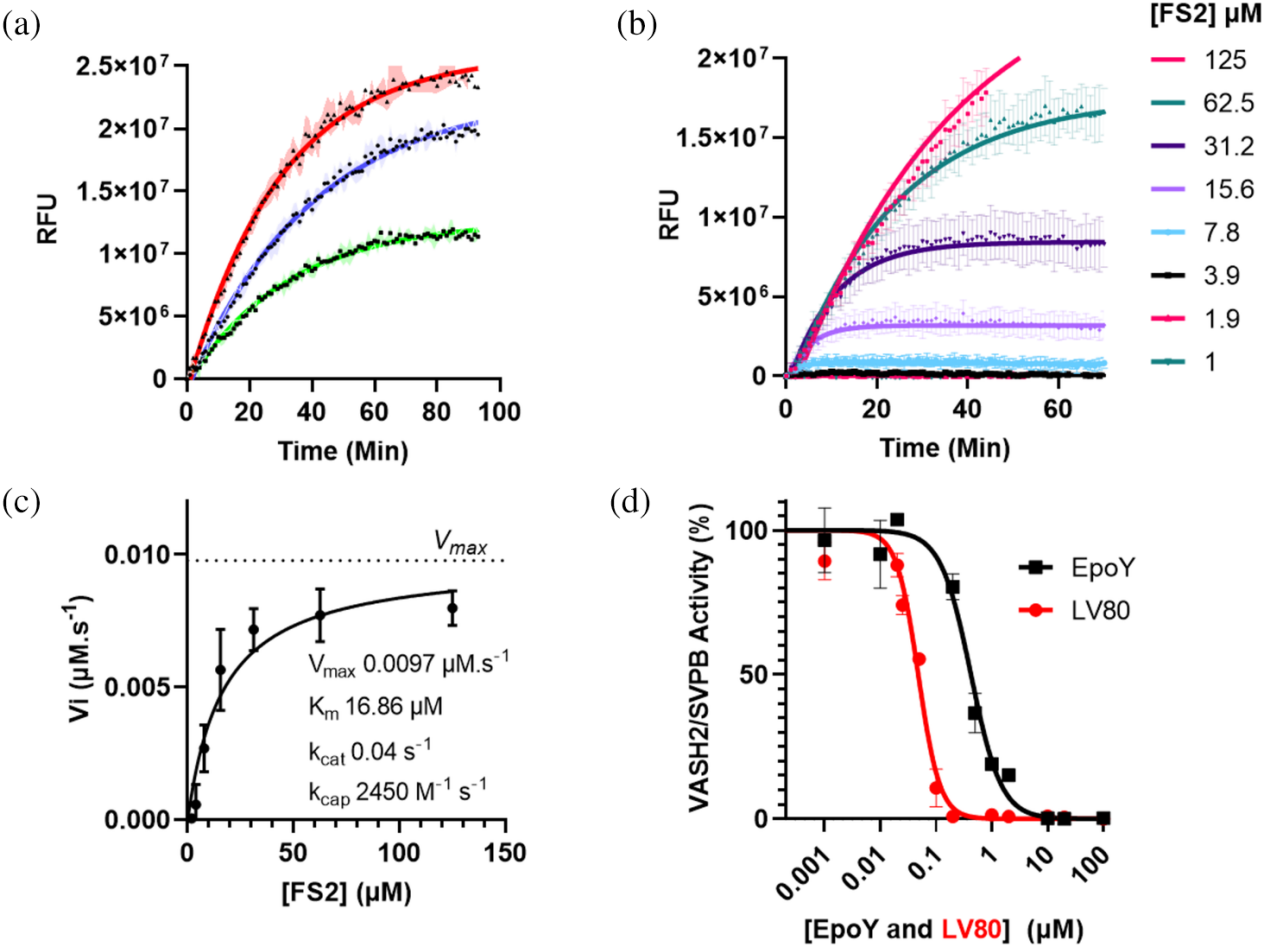
(a) Enzyme kinetics of VASH2/SVPB (235 nM) using FRET substrates (FS1‐FS2‐FS3) at 100 μM. (b) Time‐course fluorescence measurements at different substrate concentrations (FS2, 1–125 μM). (c) Initial reaction velocities (Vi) plotted against substrate concentration and fitted to the Michaelis–Menten equation using GraphPad Prism to determine kinetic parameter of VASH2/SVPB. (d) Dose–response inhibition curve of VASH2 by EpoY and LV80. Data are presented as mean ± SEM, *n* = 3 for all experiments.

Finally, after completing the study on TMCP1, we extended our investigation to two other known tubulin detyrosinases, VASH1 and VASH2, as well as to TMCP2, a paralogue of TMCP1 that cuts beta‐tubulin tail (Nicot et al., 2023), Consistent with previous reports, TMCP2 showed no detectable activity toward any of the FRET substrates reported in this study, in line with its known preference for β‐tubulin over α‐tubulin (Nicot et al., 2023). Next, we investigated the activity of VASHs in complex with their cofactor Small Vasohibin Binding Protein (SVBP). After validation of their in vitro detyrosinase activity using Sf9‐derived microtubules (Figure S2), both vasohibins were tested with the three FRET‐peptides. Despite testing multiple protein concentrations (up to 500 nM), no cleavage of the peptide substrates (FS1–3) was observed for VASH1. To rule out an effect of the nitro‐tyrosine, the assay was repeated with the NS3 peptide and analyzed by LC–MS, but again no activity was detected under these conditions. On the other hand, analogous experiments involving VASH2 revealed robust cleavage of all three peptide substrates (Figure 5a).

This striking difference suggests distinct substrate recognition by VASH1 as compared to VASH2. To achieve efficient cleavage, VASH1 may require either a longer peptide substrate or additional interactions provided by the full‐length α‐tubulin or even microtubules. However, it is important to note, that VASH1&2 active sites are structurally very similar (Feral et al., 2025). With this consideration in mind, inhibitors identified using VASH2 will have a very strong probability to also inhibit VASH1, as long as they target the active site of the enzymes. Indeed, in our previous medicinal chemistry study on VASH1&2 enzymes, the identified lead inhibitors showed high potency on both enzymes (Feral et al., 2025). The ability of VASH2, unlike TMCP1, to also hydrolyze the shortest FS1 peptide, corresponding to the three C‐terminal amino acids, reflects the distinct substrate specificities of these enzymes. Indeed, TMCP1 establishes an extensive interaction network with multiple glutamate residues, whereas substrate recognition by VASHs relies primarily on the second‐last glutamate residue (‐EEY) and the presence of an aromatic C‐terminal amino acid (Landskron et al., 2022). To ensure consistency with previous TMCP1 experiments, FS2 was used as the reference substrate. A detailed kinetic analysis was then performed using FS2 as the FRET substrate, following a workflow similar to that established for TMCP1 (Figure 5b,c). Substrate turnover was monitored in real time by recording the increase in fluorescence upon cleavage, and Michaelis–Menten parameters were determined under optimized assay conditions. Similarly to TMCP1, the cleavage of FS2 by VASH2 into FS2Δ1 and free 3‐nitrotyrosine was also confirmed by LC–MS (Figure S1). The FRET study provided a slightly lower Km for VASH2 of 16.8 μM compared to the 20.5 for TMCP1. The robustness of the assay was further validated using two VASH inhibitors we have previously reported, EpoY and LV80 (Figure 5d). A substrate concentration of 100 μM was used, similarly to the TMCP1 assay, to ensure near‐saturating conditions relative to the Km. Both compounds inhibited VASH2 activity with IC_50_ values of 0.42 and 0.048 μM for EpoY and LV80, respectively, consistent with previously reported potencies (Aillaud et al., 2017; Feral et al., 2025). This assay is specifically applicable to VASH2, however, as previously noted, inhibitors identified for VASH2 have a high likelihood of also blocking VASH1, as demonstrated by EpoY and LV80 in our earlier study (Feral et al., 2025). Taken together, these dose–response experiments with the two model inhibitors confirm the robustness of the FRET assay for assessing VASH2 inhibitors, and indirectly also support its relevance for VASH1, as long as the inhibitors target the enzymes' active sites.

## 4 CONCLUSIONS

The development and validation of the FRET‐based enzymatic assay for TMCP1 provides a robust, site‐specific, and reliable method for monitoring enzymatic activity and screening of potential inhibitors. While it may not detect inhibitors that interfere with the enzyme‐microtubule interface, it offers a straightforward and effective approach for identifying active‐site‐directed inhibitors. A key innovation of this approach lies in the use of 3‐nitrotyrosine as a quencher, which not only restores fluorescence upon enzymatic cleavage but also closely mimics the natural tyrosine substrate at position P1'. Our strategy ensures optimal recognition by TMCP1 while preserving the essential terminal carboxyl group required for metallocarboxypeptidase activity, making it particularly well‐suited for this class of enzymes.

The FS2 fluorogenic substrate was selected for its high quenching efficiency combined with effective enzymatic processing. Kinetic analysis revealed that TMCP1 exhibits moderate activity with FS2, yet the assay conditions provide a reliable and practical framework for enzymatic measurements. Key experimental controls, including competition assays with the natural substrate (NS3) and inhibition assays with EDTA, confirmed both the specificity of the substrate and the robustness of the assay. Additionally, the metal ion dependency study demonstrated TMCP1's preference for Co^2+^ and Mn^2+^ over Zn^2+^, raising questions about the physiologically relevant metal ion used by the protease in vivo.

Further optimization of buffer conditions, ionic strength, and pH ensured the assay's reproducibility across a range of experimental settings. The determination of an IC_50_ for EDTA, consistent with previous reports (Lavrsen et al., 2023), validated the assay's suitability for inhibitor screening. With the ability to precisely quantify enzymatic activity and inhibition under physiologically relevant conditions, this assay represents a powerful tool for drug discovery targeting TMCP1.

In addition, we extended the assay to VASH2, another protease catalyzing detyrosination, and established that this enzyme was able to process all three FRET peptides (FS1‐3). This finding significantly increases the relevance of the assay, as it can now be applied to the development of inhibitors targeting two of the three known enzymes with α‐tubulin detyrosinase activity. Interestingly, VASH1 was not able to process the FRET peptides suggesting that this enzyme requires a more complex interaction with the microtubules. Collectively, these observations reinforce the notion that, despite TMCP1, VASH1, and VASH2 sharing the same detyrosination activity, their distinct substrate preferences argue against functional redundancy. Rather, they suggest specialized and potentially non‐overlapping roles in the cellular context, underscoring an additional layer of regulatory control in microtubule dynamics and tubulin posttranslational modifications. The assay we established here provides a foundation for developing VASH‐ or TMCP1‐specific inhibitors, as well as broad microtubule detyrosination inhibitors. Such chemical tools will be invaluable in elucidating the role of each class of detyrosinases. Furthermore, accumulating evidence supports the critical role of tubulin posttranslational modifications in both neurodegeneration and cancer. In this context, the selective modulation of microtubule dynamics through enzyme inhibition holds significant therapeutic potential.

## AUTHOR CONTRIBUTIONS

**Matthieu Simon:** Conceptualization; investigation; methodology; validation; formal analysis; writing – original draft; writing – review and editing; data curation; visualization. **Julien Espeut:** Writing – review and editing; investigation; formal analysis. **François Juge:** Investigation; writing – review and editing; formal analysis; methodology. **Muriel Amblard:** Writing – review and editing; funding acquisition; supervision. **Krzysztof Rogowski:** Conceptualization; funding acquisition; writing – review and editing; resources; supervision; validation. **Lubomir Vezenkov:** Supervision; resources; conceptualization; funding acquisition; writing – review and editing; validation.

## Supporting information


**DATA S1.** Supporting Information.

## Data Availability

The data that support the findings of this study are available from the corresponding author upon reasonable request.
