# Supplementary material for: Real‐time FRET assay for monitoring detyrosination by TMCP1 and VASH2
Source: Protein Sci. 2025 Nov 12;34(12):e70374. doi: 10.1002/pro.70374 (PMC12612596; doi:10.1002/pro.70374)
Supplement: Supplementary file 1 — DATA S1. Supporting Information. [file PRO-34-e70374-s001.docx]

**Real-Time FRET Assay for Monitoring Detyrosination by TMCP1 and VASH2**

**Supporting Information**

**Matthieu Simon^1^, Julien Espeut^2^, François Juge^2^ Muriel Amblard^1^, Krzysztof Rogowski^2^*, Lubomir Vezenkov^1^***

*- corresponding authors and equal contribution

1. IBMM, Université Montpellier, CNRS, ENSCM, Montpellier, France.
2. Tubulin Code team, Institute of Human Genetics, Université Montpellier, CNRS, Montpellier, France


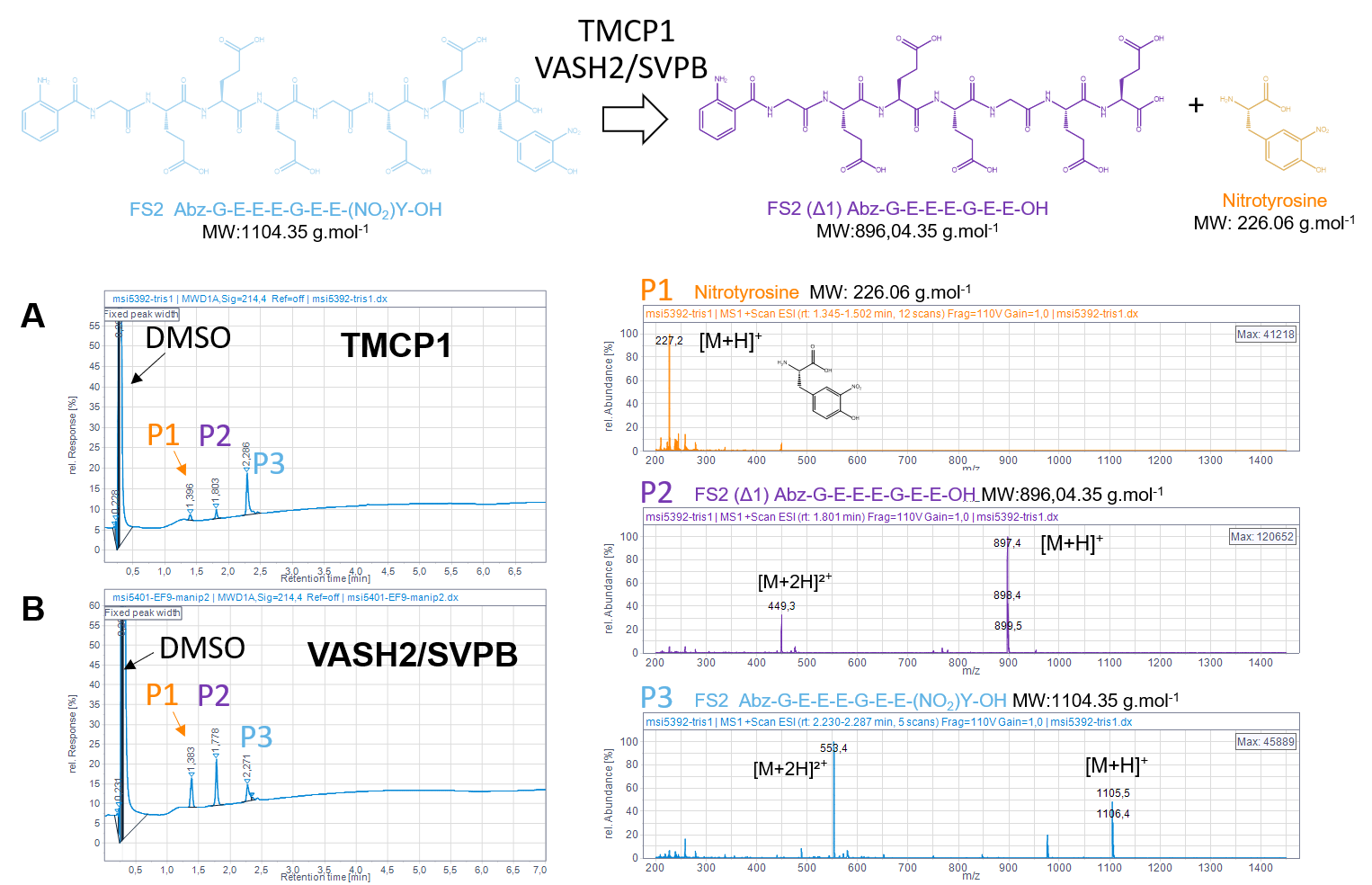


**Fig. S1.** **A**) LC-MS analysis after 20 min incubation of FS2 (100 µM) with TMCP1 (120 nM) at 37 °C in 50 mM Tris-HCl buffer (pH 7.4), **B**) LC-MS analysis after 90 min incubation of FS2 (100 µM) with VASH2/SVPB (235 nM) at 37 °C in 50 mM Tris-HCl buffer (pH 7.4), confirming the cleavage of FS2 into FS2Δ1 and free 3-nitrotyrosine3-nitrotyrosine by both enzymes (upper panel). Three similar peaks (P1–P3) are detected on both chromatogram A and B (UV 214 nm, left panel), corresponding respectively to free 3-nitrotyrosine (P1), FS2Δ1 (P2), and intact FS2 (P3). The differences in peak intensity reflect the extent of reaction progress, in the experiment with VASH2, nearly all of the peptide substrate was cleaved. The identity of the molecules under each peak is confirmed by the corresponding mass spectra (right panel, positive ion mode), which reveal FS2 and FS2Δ1 Detected as [M+H]⁺ and [M+2H]²⁺, while 3-nitrotyrosine is observed only as the singly charged [M+H]⁺.LC-MS analysis was carried out by liquid chromatography (Agilent 1290 Infinity II) coupled to a high-resolution mass spectrometer (Agilent LC/MSD iQ), equipped with an electrospray ionization source and controlled by OpenLab.


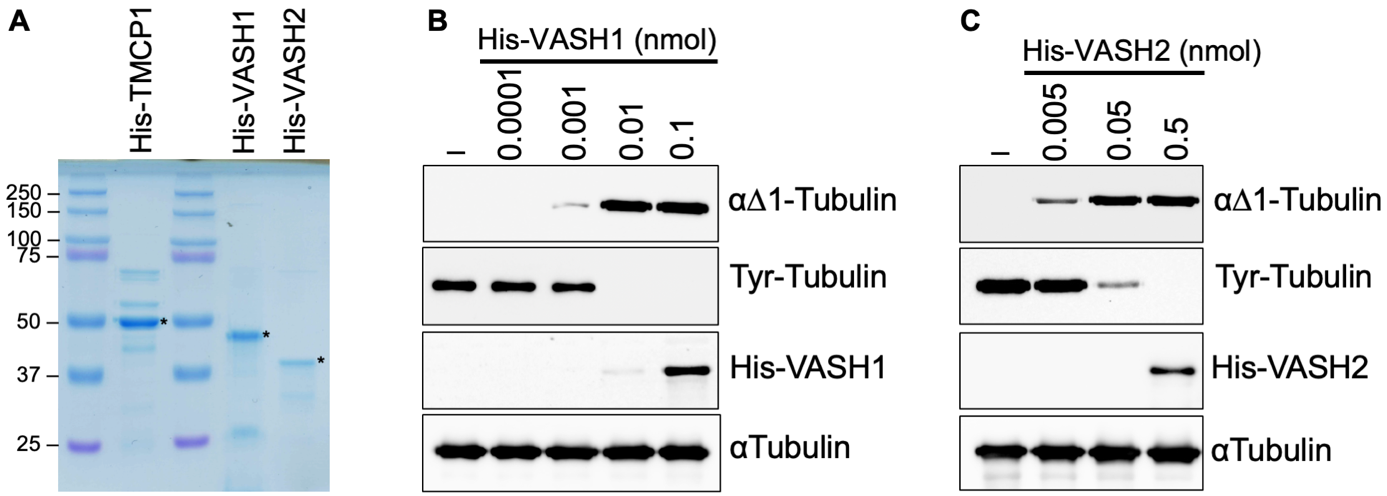


**Fig. S2. A) Coomassie stained gel demonstrating the purity of the three enzymes used in the *in vitro* assays.** The bands corresponding to purified His-tagged proteins are indicated by an asterisk. **B, C) Immunoblots confirming the *in vitro* detyrosinase activity of VASH1/SVBP and VASH2/SVBP on microtubules.** *In vitro* detyrosination assays were performed with 2 µg of Sf9-derived MTs during 30 min at 37° in 40 µl of buffer (50 mM Tris pH 7.4, 20 µM taxol, 10% glycerol) with the indicated amount of His-VASH1/SVBP (B) or His-VASH2/SVBP (C).
